# A screening identifies harmine as a novel antibacterial compound against *Ralstonia solanacearum*

**DOI:** 10.3389/fmicb.2023.1269567

**Published:** 2023-09-05

**Authors:** Hongkai Xia, Yanxia Huang, Ruoyu Wu, Xin Tang, Jun Cai, Shun-xiang Li, Lin Jiang, Dousheng Wu

**Affiliations:** ^1^Hunan Key Laboratory of Plant Functional Genomics and Developmental Regulation, College of Biology, Hunan University, Changsha, China; ^2^Research Institute of HNU in Chongqing, Chongqing, China; ^3^Department of Pathology and Pathophysiology, School of Medicine, Jishou University, Jishou, China; ^4^Hunan Engineering Technology Research Center for Bioactive Substance Discovery of Chinese Medicine, School of Pharmacy, Hunan University of Chinese Medicine, Changsha, China

**Keywords:** *Ralstonia solanacearum*, bacterial wilt, harmine, plant-derived compound, disease control

## Abstract

*Ralstonia solanacearum*, the causal agent of bacterial wilt, is a devastating plant pathogenic bacterium that infects more than 450 plant species. Until now, there has been no efficient control strategy against bacterial wilt. In this study, we screened a library of 100 plant-derived compounds for their antibacterial activity against *R. solanacearum*. Twelve compounds, including harmine, harmine hydrochloride, citral, vanillin, and vincamine, suppressed bacterial growth of *R. solanacearum* in liquid medium with an inhibition rate higher than 50%. Further focus on harmine revealed that the minimum inhibitory concentration of this compound is 120 mg/L. Treatment with 120 mg/L of harmine for 1 and 2 h killed more than 90% of bacteria. Harmine treatment suppressed the expression of the virulence-associated gene *xpsR*. Harmine also significantly inhibited biofilm formation by *R. solanacearum* at concentrations ranging from 20 mg/L to 60 mg/L. Furthermore, application of harmine effectively reduced bacterial wilt disease development in both tobacco and tomato plants. Collectively, our results demonstrate the great potential of plant-derived compounds as antibacterial agents against *R. solanacearum*, providing alternative ways for the efficient control of bacterial wilt.

## Introduction

*Ralstonia solanacearum*, the causal agent of bacterial wilt, is a devastating soil-borne bacterium capable of causing wilting disease in plants (Álvarez et al., 2010). Being a species complex, *R. solanacearum* has a wide host range and can infect over 450 plant species (Ahmed et al., 2022). Under favorable conditions, the bacterium can survive for extended periods in soil, plant debris, and freshwater (Álvarez et al., 2010; Caldwell et al., 2017). Upon sensing plant signals, *R. solanacearum* exhibits chemotaxis, moving toward the plant roots using its flagella, and then infects the plant through wounds or natural openings. Once inside the roots, *R. solanacearum* rapidly multiplies in the xylem and secretes large amounts of extracellular polysaccharides that clog the plant’s water and nutrient-conducting xylem vessels when the bacterial population is well established (Tran et al., 2016; She et al., 2017). This ultimately leads to wilting of the entire plant, known as bacterial wilt. Due to its significance academic impact and devastating economic losses, *R. solanacearum* is recognized as the second most important bacterial pathogen (Mansfield et al., 2012).

Control measures for bacterial wilt are crucial to minimize its devastating impact on plant growth (Hayward, 1991). Currently, agricultural practices mainly include methods such as soil disinfection, grafting, crop rotation, resistance breeding, and the use of chemical or biological agents to control bacterial wilt (Ahmed et al., 2022). Soil disinfection techniques, such as solarization and fumigation, aim to reduce the pathogen population in the soil (Ahmed et al., 2022). Crop rotation involves alternating susceptible and resistant crops to interrupt the pathogen’s life cycle (Fan et al., 2022). Breeding programs focus on developing resistant varieties through conventional breeding or genetic engineering (Genin and Denny, 2012; Safni et al., 2014). While certain control measures have exhibited a degree of effectiveness, they frequently do not meet the anticipated outcomes. Although the use of molecular markers to identify resistance genes in hosts of *R. solanacearum* has greatly facilitated the breeding of resistant varieties, challenges remain in stabilizing the inheritance of resistance genes and determining the impact of resistant varieties on yield (Qian et al., 2013; Nelson et al., 2018; Abebe et al., 2020). Chemical pesticides can effectively eradicate *R. solanacearum*; nevertheless, they not only induce environmental pollution but also foster the development of pesticide resistance within the bacterium (Nicolopoulou-Stamati et al., 2016). Therefore, it is crucial to develop safe, environmentally friendly, efficient, and economically viable strategies for controlling bacterial wilt.

Natural products refer to bioactive secondary metabolites isolated from animals, plants, microorganisms, and marine organisms. They offer advantages such as low cost, high safety, and effectiveness (Shen and Hao, 2020). In recent years, many natural products have not only been applied in the prevention and treatment of bacterial plant diseases but also serve as lead compounds for biopesticides (Zhang et al., 2018). Plant-derived compounds, specifically New 8-O-4’ Neolignans isolated from the whole plant of *Clematis lasiandra* Maxim, have been found to inhibit various plant pathogens, including *R. solanacearum* (Hao et al., 2020). Coumarins, secondary metabolites derived from plants, have also been discovered to significantly inhibit *R. solanacearum*, with enhanced activity through hydroxylation at the C-6, C-7, and C-8 positions of the coumarin structure (Yang et al., 2021b).

Harmine, also known as telepathine, is a natural alkaloid found in several plants, including *Peganum harmala L.* and *Banisteriopsis caapi* (Callaway et al., 1999). It has gained considerable attention due to its potential therapeutic effects and wide-ranging pharmacological activities. Research suggests that harmine exhibits a range of effects on the central nervous system (dos Santos and Hallak, 2017). Harmine has been found to act as a reversible inhibitor of monoamine oxidase-A (MAO-A), an enzyme involved in the breakdown of neurotransmitters such as serotonin, norepinephrine, and dopamine (Dakic et al., 2016; dos Santos and Hallak, 2017). By inhibiting MAO-A, harmine can increase the levels of these neurotransmitters, leading to mood enhancement and potential antidepressant effects (Dakic et al., 2016). Moreover, harmine has been investigated for its impact on cognitive function. Studies have indicated its potential as a neuroprotective agent, with the ability to enhance memory and improve learning abilities (Zhang et al., 2020). Additionally, harmine has been explored for its anti-inflammatory, antioxidant, and anticancer properties, showing promising results in various preclinical studies. However, the effect of harmine on plant pathogenic bacteria, such as *R. solanacearum*, remains unknown.

In this study, we identified harmine as a promising antibacterial agent against *R. solanacearum*. We found that harmine has a minimum inhibitory concentration of 120 mg/L. Biofilm formation of *R. solanacearum* was also significantly suppressed by different concentrations of harmine. Application of harmine effectively delayed bacterial wilt disease development in both tobacco and tomato plants.

## Materials and methods

### Bacterial strains and growth conditions

The *R. solanacearum* strain CQPS-1, which was originally isolated from diseased tobacco plants in Pengshui, Chongqing, was used in this study (Liu et al., 2017). *R. solanacearum* was grown in B liquid medium (1% peptone, 0.1% tryptone, 0.1% yeast extract, 2.5% glucose) or B solid medium supplemented with 1.5% agar at 28°C.

### Compounds information

All plant-derived compounds (HPLC >98%) used in this study were purchased from Targetmol (Shanghai, China). Compounds were dissolved in dimethyl sulfoxide (DMSO) to a final concentration of 10 mM or 100 mM and stored at-20°C. Ten minutes before use, the frozen compound solutions were thawed at room temperature and added to B or B agar medium to designed concentrations. The same volume of DMSO was used as solvent control.

### Screening of bioactive antibacterial compounds

The screening of bioactive antibacterial compounds against *R. solanacearum* was carried out in 1.5 mL tubes. Briefly, 282 μL of B liquid medium was added to individual tube, followed by adding 15 μL of OD_600_ = 0.01 bacterial suspension. Then 3 μL of 10 mM compound solution was added to the culture mixture to reach the final concentration of 100 μM. The same volume of DMSO was used as solvent control. The culture mixture was incubated in a shaker with 180 rpm at 28°C. The OD_600_ was measured 24 h post-incubation. Three technical replicates were used for each treatment. The inhibitory rate was calculated by the following formula, inhibitory rate (%) = (OD_600_ of DMSO treated samples − OD_600_ of compound treated samples) / OD_600_ of DMSO treated samples  × 100%.

### Determination of growth curve of *Ralstonia solanacearum*

The growth curve of *R. solanacearum* was determined as follows. Briefly, 25 μL of OD_600_ = 0.1 bacterial suspension was added into 5 mL of B liquid medium, followed by adding different amounts of harmine to reach the final concentration of 20 mg/L, 40 mg/L, 60 mg/L, 80 mg/L or 100 mg/L, respectively. The same volume of DMSO was used as solvent control. The culture mixture was incubated in a shaker with 180 rpm at 28°C. The OD_600_ was measured every 2 h post-incubation. Three technical replicates were used for each treatment.

### Determination of MIC

The minimum inhibitory concentration (MIC) was determined using the dilution method with a series of harmine concentrations ranging from 20 mg/L to 120 mg/L. The OD_600_ of the *R. solanacearum* suspension was adjusted to 0.01 with sterile water. Next, 15 μL of the OD_600_-adjusted bacterial suspension was added into 285 μL of liquid B medium containing different concentrations of harmine, ranging from 20 mg/L to 120 mg/L. The inoculated culture was then incubated at 28°C for 24 h. The MIC was defined as the lowest concentration at which no visible growth of *R. solanacearum* was observed at 48 h post-incubation.

The inhibitory effect of harmine on the growth of *R. solanacearum* in solid medium was evaluated as follows. Harmine was added into B solid medium to reach final concentrations ranging from 40 mg/L to 340 mg/L. Then, 50 μL of OD_600_ = 0.0001 bacterial suspension were plated on B solid medium supplemented with different concentrations of harmine. The bacterial growth was observed at 2 days post-incubation.

### Bactericidal activity assay

The overnight-cultured *R. solanacearum* suspension was centrifuged and re-suspended in sterile water. The OD_600_ was adjusted to 0.1. Then, the bacterial suspension was divided into 15 mL centrifuge tubes, with 3 mL in each tube. Harmine was added to each tube to generate final concentrations of 60, 120, 180, or 240 mg/L. The control group was treated with an equal amount of DMSO. The harmine-treated bacterial suspension was then incubated at 28°C. At 1 h or 2 h post-incubation, 100 μL of the incubated suspension was sampled and diluted to create 10^−1^, 10^−2^, 10^−3^, 10^−4^, and 10^−5^ dilutions. Three drops of 5 μL of each diluted bacterial suspension were then placed on B agar plates. The B agar medium was incubated at 28°C for 48 h. Live bacteria were counted, and the sterilizing rate was calculated.

### Biofilm formation analysis

Biofilm formation was analyzed as previously described with some modifications (Wu et al., 2015). Briefly, 10 μL of *R. solanacearum* suspension (OD_600_ = 0.1) was added to 190 μL of liquid medium B in a 96-well polystyrene microplate. Harmine was then added to each well to reach final concentrations of 20 mg/L, 40 mg/L, or 60 mg/L. DMSO was used as the negative control. The 96-well plate was placed in an incubator at 28°C for static cultivation for 24 h. After incubation, the culture medium was gently aspirated using a micropipette, followed by gentle washes with 200 μL of sterile water to remove the medium. Next, 220 μL of 0.1% crystal violet was added and the plate was stained at room temperature for 30 min. After staining, the crystal violet was aspirated, and the wells were washed twice with 200 μL of sterile water to remove excess dye. The plate was air-dried at room temperature for 30 min, and then 200 μL of 95% ethanol was added to dissolve the crystal violet adsorbed on the biofilm. Finally, the 96-well plate was placed in a microplate reader to measure the absorbance at 530 nm.

### Quantitative real-time PCR

The effect of harmine on the expression of virulence-related genes of *R. solanacearum* was tested using quantitative real-time PCR (qPCR), following the procedure previously described with minor modifications (Wu et al., 2015). Fifty microliters of *R. solanacearum* suspension (OD_600_ = 1.0) was added to 5 mL of liquid medium, and harmine was added to reach a final concentration of 80 mg/L. The mixture was then incubated at 28°C for 6 h. The bacterial cells were harvested by centrifugation, and the total RNA of *R. solanacearum* was extracted using the Unizol Total RNA Extraction Reagent, following the manufacturer’s instructions (Genesand). Subsequently, cDNA was synthesized with the HiScript II 1st Strand cDNA Synthesis Kit, following the protocol provided by the company (Vazyme). For qPCR, 20 μL reactions (BioRad, CFX96) were prepared, each containing 1 pmol of forward and reverse primers, 2 μL of 1 to 10 diluted cDNA, and 4 μL of ChamQ Universal SYBR qPCR Master Mix (Vazyme). The primers used were the same as previously described (Han et al., 2021). Relative gene expression was calculated using the 2^-∆∆CT^ method.

### Inoculation assay

The naturalistic soil soak assay was used to evaluate the effectiveness of harmine in controlling tobacco and tomato bacterial wilt. Briefly, 4-week-old tobacco and tomato plants were first inoculated with 10 mL of *R. solanacearum* suspension (OD_600_ = 0.1). One hour later, the seedlings were treated with 10 mL of harmine at a concentration of 150 mg/L. An equal volume of diluted DMSO was used as the negative control. The inoculated plants were then placed in a growth chamber at 28°C with a 14/10 h light/dark photoperiod. The symptoms were scored daily using a disease index scale ranging from 0 to 4 (0, no symptoms appeared, 1, 1 to 25% of leaves wilted, 2, 26 to 50% of leaves wilted; 3, 51 to 75% of leaves wilted; 4, 76 to 100% of leaves wilted). The control efficiency was calculated with the following formula: Control efficiency % = (Disease index of DMSO treated seedlings – Disease index of harmine treated seedlings) / Disease index of DMSO treated seedlings  × 100%.

### Statistical analysis

The data were analyzed with either Excel or GraphPad Prism using Student’s *t*-test (*p < 0.05, **p < 0.01, ***p < 0.001, ****p < 0.0001).

## Results

### A screen identifies several plant-derived compounds as antibacterial agents against *Ralstonia solanacearum*

To identify new antibacterial agents against *R. solanacearum*, we tested the impact of 100 plant-derived compounds on its growth in liquid medium at a concentration of 100 μM. To quantify the antibacterial activity of each compound, we used the inhibitory rate, which refers to the percentage reduction in bacterial growth (the OD_600_ value) as a result of the treatment with a specific compound. Twelve compounds, namely harmine, harmine hydrochloride, citral, vanillin, vincamine, anisodamine, decitabine, higenamine hydrochloride, solanesol, alpha-Boswellic acid, and trimethoxystilbene, exhibited a very strong inhibitory effect on the growth of *R. solanacearum*, with an inhibitory rate higher than 50% (Table 1). Among these bioactive compounds, harmine showed the most potent inhibitory effect. The average OD_600_ of the DMSO-treated sample, which served as the solvent control, was 1.19, while the average OD_600_ of the harmine-treated sample was 0.25 (Table 1), indicating a significant suppression of *R. solanacearum* growth by harmine in liquid medium. Some other compounds, such as eburnalritardo and chlorogenic acid, also exhibited inhibitory effects on the growth of *R. solanacearum*, though the inhibitory rate was not as strong as that of the 12 compounds mentioned above (Table 1). Collectively, the screening identified several plant-derived compounds as novel antibacterial agents against *R. solanacearum*.

**Table 1:** Screening of antimicrobial compounds against *R. solanacearum.*

Number	Chemical	OD_600_	Number	Chemical	OD_600_
	DMSO	1.11 ± 0.05		DMSO	1.19 ± 0.06
1	Monocrotaline	0.99 ± 0.05	51	Madecassoside	1.13 ± 0.05
2	Rhoifolin	1.07 ± 0.09	52	Bilobalide	1.04 ± 0.07
3	Genipin	1.18 ± 0.04	53	Etoposide	0.80 ± 0.08
4	Harmine hydrochloride	0.44 ± 0.04	54	Galanthamine HBr	1.08 ± 0.08
5	Schisantherin A	1.04 ± 0.13	55	Anisodamine	0.47 ± 0.08
6	Pectolinarin	0.85 ± 0.05	56	(+)-Shikonin	0.62 ± 0.04
7	Deacetyltaxol	0.92 ± 0.03	57	Lanatoside C	1.09 ± 0.08
8	Saikosaponin A	0.98 ± 0.09	58	Harmine	0.25 ± 0.04
9	Picroside I	1.09 ± 0.05	59	Decitabine	0.48 ± 0.05
10	Ginsenoside Rg2	0.99 ± 0.03	60	Pseudolaric Acid B	0.70 ± 0.08
11	Betulin	0.85 ± 0.04	61	Euphorbiasteroid	0.67 ± 0.12
12	Echinacoside	0.98 ± 0.09	62	Citral	0.42 ± 0.04
13	(+)-Fangchinoline	1.01 ± 0.09	63	Dihydroactinidiolide	1.04 ± 0.04
14	Arteether	1.08 ± 0.06	64	Sophoricoside	1.03 ± 0.08
15	Salvianolic Acid C	0.89 ± 0.06	65	Higenamine Hydrochloride	0.46 ± 0.09
16	Caudatin	0.96 ± 0.10	66	Solanesol	0.59 ± 0.03
17	Abietic Acid	1.06 ± 0.11	67	Aloperine	1.04 ± 0.09
18	Harmol	0.96 ± 0.04	68	Tabersonine	0.92 ± 0.06
19	Silymarin	1.03 ± 0.13	69	Citronellol	0.94 ± 0.08
20	Eburnalritardo	0.61 ± 0.10	70	(S)-(+)-Carvone	1.03 ± 0.09
21	Vincamine	0.45 ± 0.03	71	Stachydrine Hydrochloride	0.65 ± 0.09
22	10-Deacetylbaccatin III	0.97 ± 0.09	72	alpha-Boswellic acid	0.58 ± 0.06
23	S-Isocorydine(+)	0.70 ± 0.09	73	Geranyl Tiglate	1.05 ± 0.08
24	Orcinol glucoside	1.13 ± 0.08	74	Vanillin	0.45 ± 0.05
25	(20S)-Protopanaxatriol	0.64 ± 0.04	75	Perillyl alcohol	0.51 ± 0.08
26	Ginkgolide B	1.08 ± 0.08	76	Oleanonic Acid	0.54 ± 0.02
27	Tangeretin	1.17 ± 0.05	77	Parthenolide	0.90 ± 0.08
28	Forsythin	0.94 ± 0.09	78	Trimethoxystilbene	0.48 ± 0.05
29	Paclitaxel	0.88 ± 0.05	79	Lycorine	1.05 ± 0.11
30	Dehydrocostus Lactone	1.00 ± 0.10	80	Angelic Acid	1.08 ± 0.04
31	Azelaic acid	1.08 ± 0.04	81	(+)-Thalrugosine	1.13 ± 0.07
32	Panthenol	1.55 ± 0.18	82	Scopoletin	0.81 ± 0.05
33	4-Methylumbelliferone	0.74 ± 0.08	83	Trigonelline	1.46 ± 0.06
34	Sucralose	1.75 ± 0.07	84	Cycloastragenol	1.20 ± 0.04
35	L-Menthol	1.24 ± 0.04	85	Schizandrin B	1.31 ± 0.11
36	Khellin	1.23 ± 0.06	86	Ginsenoside Rb1	1.66 ± 0.08
37	8-Methoxypsoralen	1.18 ± 0.11	87	Daidzin	1.49 ± 0.07
38	Resveratrol	1.09 ± 0.08	88	Psoralen	1.28 ± 0.03
39	Aloin	1.39 ± 0.03	89	Astilbin	1.55 ± 0.09
40	Aloe-emodin	1.22 ± 0.04	90	Isorhamnetin	1.38 ± 0.09
41	Chrysin	0.99 ± 0.07	91	Hydroxyecdysone	1.03 ± 0.08
42	Eupatilin	1.19 ± 0.05	92	Arctiin	1.58 ± 0.04
43	Aminophylline	1.18 ± 0.11	93	Icariin	0.77 ± 0.05
44	Hematoxylin	1.15 ± 0.09	94	Rosmarinic acid	1.61 ± 0.06
45	Vinblastine sulfate	1.29 ± 0.11	95	Patchouli alcohol	1.59 ± 0.09
46	Cabazitaxel	1.40 ± 0.03	96	Arctigenin	1.06 ± 0.13
47	Bavachinin	1.04 ± 0.05	97	Chlorogenic Acid	0.65 ± 0.08
48	Kaempferol	0.99 ± 0.04	98	Catalpol	0.94 ± 0.05
49	Eleutheroside E	1.16 ± 0.07	99	Schisandrin	1.58 ± 0.04
50	Astragaloside IV	0.91 ± 0.05	100	Curcumol	1.13 ± 0.08

### Harmine inhibits the growth of *Ralstonia solanacearum* in a concentration dependent manner

**Figure 1:**
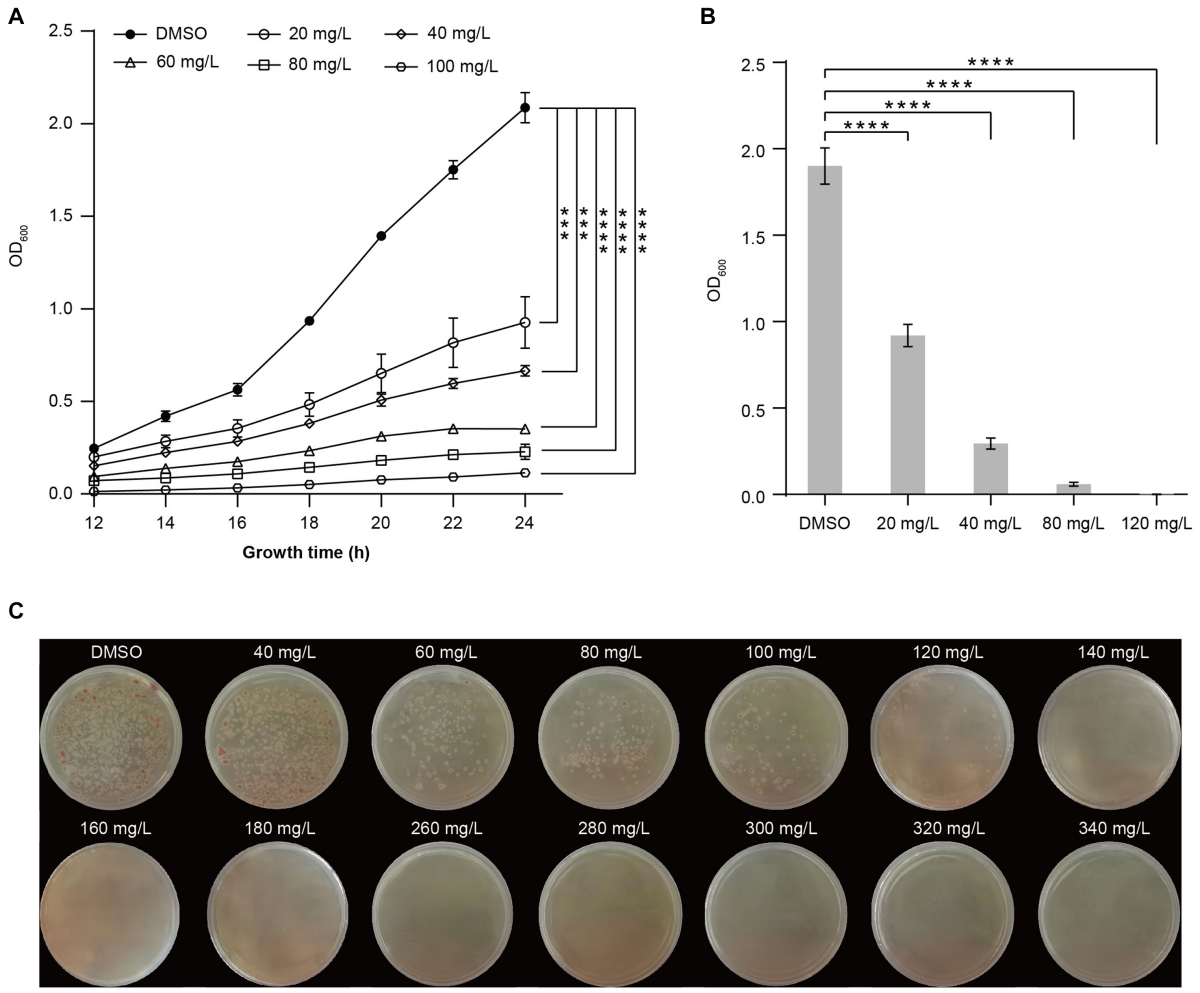
Harmine inhibits the growth of *R. solanacearum* in a concentration-dependent manner. **(A)** The growth curve of *R. solanacearum* in liquid medium supplemented with DMSO or different concentrations of harmine. The OD_600_ was measured every 2 h starting from 12 h post-incubation. **(B)** The inhibitory effect of different concentrations of harmine on *R. solanacearum* growth at 24 h post-incubation. Data are presented as the mean ± SD (*n* = 3). ***p < 0.001, ****p < 0.0001 (Student’s *t*-test). **(C)** Inhibition of *R. solanacearum* growth on B agar plate supplemented with different concentrations of harmine. B agar plate was supplemented with 0.005% triphenyl tetrazolium chloride (TTC). Pictures were taken 48 h post-incubation.

To further determine the impact of harmine on inhibiting *R. solanacearum* growth, we tested the inhibitory effect of different concentrations of harmine, ranging from 20 mg/L to 100 mg/L. The growth curve from 12 h to 24 h indicated that all tested concentrations had a strong inhibitory effect on the growth of *R. solanacearum* (Figure 1A). The inhibitory rate reached about 90% at 100 mg/L and decreased with the reduction of harmine concentration. At 24 h post-treatment, the inhibitory rate decreased to about 50% at 20 mg/L (Figures 1A,B). The growth of *R. solanacearum* was completely suppressed when the liquid medium was supplemented with 120 mg/L of harmine (Figure 1B). Therefore, this concentration was defined as the MIC of harmine against *R. solanacearum*. We further confirmed the inhibitory effect of harmine against *R. solanacearum* using the solid dilution method. The result showed that supplementation of harmine into the agar plate significantly suppressed the growth of *R. solanacearum* at 120 mg/L or higher (Figure 1C).

**Figure 2:**
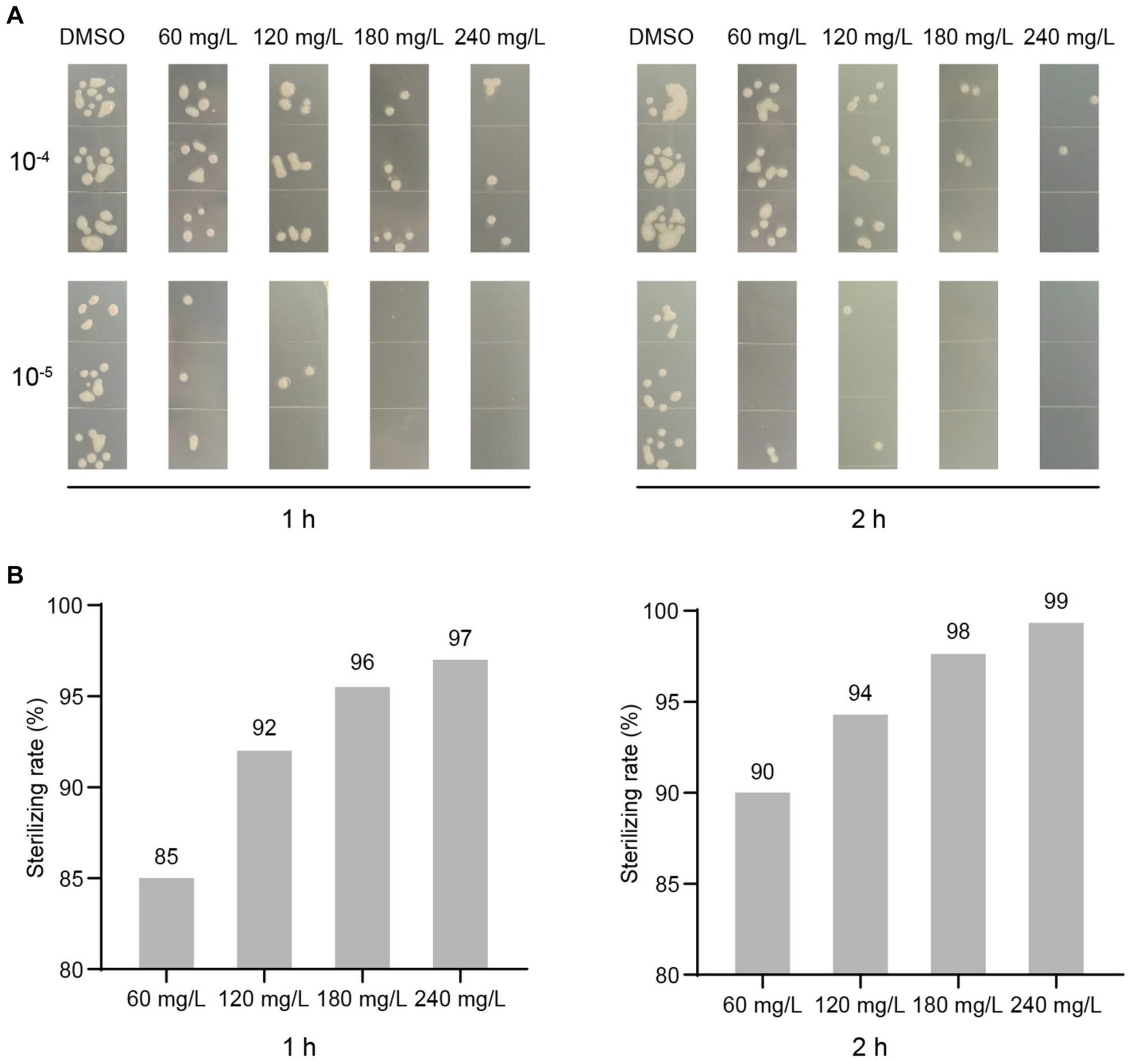
Harmine exhibits bactericidal activity against *R. solanacearum*. The OD_600_ of the *R. solanacearum* suspension was adjusted to 0.1. DMSO and different concentrations of harmine were added to the *R. solanacearum* suspension. The harmine-treated bacterial suspension was then incubated at 28°C. At 1 h and 2 h post-treatment, the bacterial suspension was diluted, plated on agar medium, and further incubated at 28°C for 2 days. **(A)** The live bacteria that grew on agar plates from dilutions of 10^−4^ and 10^−5^ were shown. **(B)** The live bacteria from dilutions of 10^−4^ were counted, and the sterilizing rates were calculated, with DMSO-treated samples as the background.

To determine if harmine exhibits bactericidal activity, we treated *R. solanacearum* suspension with different concentrations of harmine, using DMSO as the negative control. At 1 h and 2 h post-treatment, bacterial suspensions were diluted and plated on agar plates for live bacteria counting. Treatment with 60 mg/L of harmine resulted in the death of 85 and 90% of bacteria at 1 h and 2 h, respectively (Figure 2). When 180 mg/L of harmine was applied, 96 and 98% of bacteria were killed at 1 h and 2 h, respectively (Figure 2). Furthermore, at 2 h post-treatment with 240 mg/L of harmine, 99.3% of bacteria were killed (Figure 2). These results strongly suggest that harmine possesses bactericidal activity against *R. solanacearum*.

### Harmine reduces the biofilm formation by *Ralstonia solanacearum*

**Figure 3:**
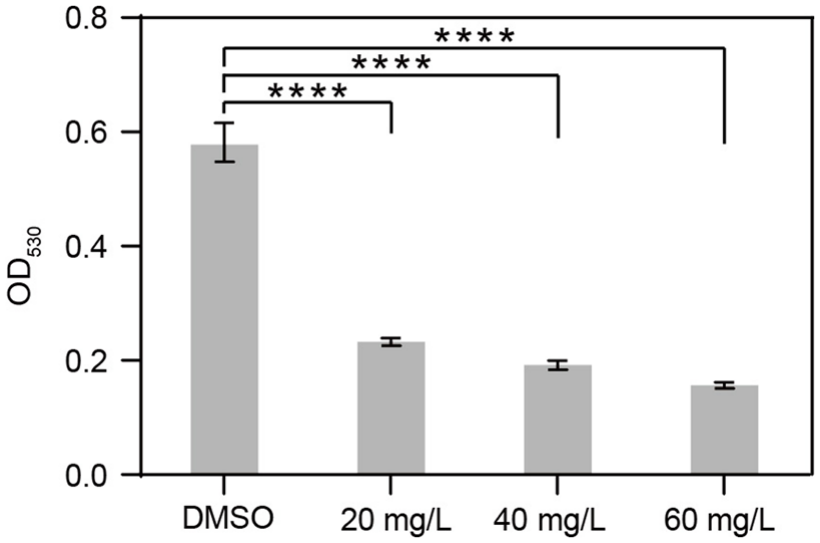
Harmine reduces biofilm formation by *R. solanacearum*. *R. solanacearum* was grown in liquid medium supplemented with different concentrations of harmine for 24 h and then subjected to biofilm quantification. Data are presented as the mean ± SD (*n* = 3). ****p < 0.0001 (Student’s *t*-test).

To evaluate the effect of harmine on biofilm formation by *R. solanacearum*, we utilized the standard polyvinyl chloride (PVC) assay to quantify biofilm in the absence or presence of harmine, with concentrations ranging from 20 mg/L to 60 mg/L. Compared to the DMSO treatment, supplementation with harmine significantly reduced biofilm formation by *R. solanacearum*. When 20 mg/L and 60 mg/L of harmine were applied, the biofilm formation was reduced by 59 and 72%, respectively (Figure 3). Notably, the reduction in biofilm formation was similar when different concentrations (20 mg/L, 40 mg/L, and 60 mg/L) of harmine were applied (Figure 3), despite that statistical significance was also detected between 20 mg/L and 40 mg/L treatment.

### Harmine suppresses the expression of the virulence-related gene *xpsR*

**Figure 4:**
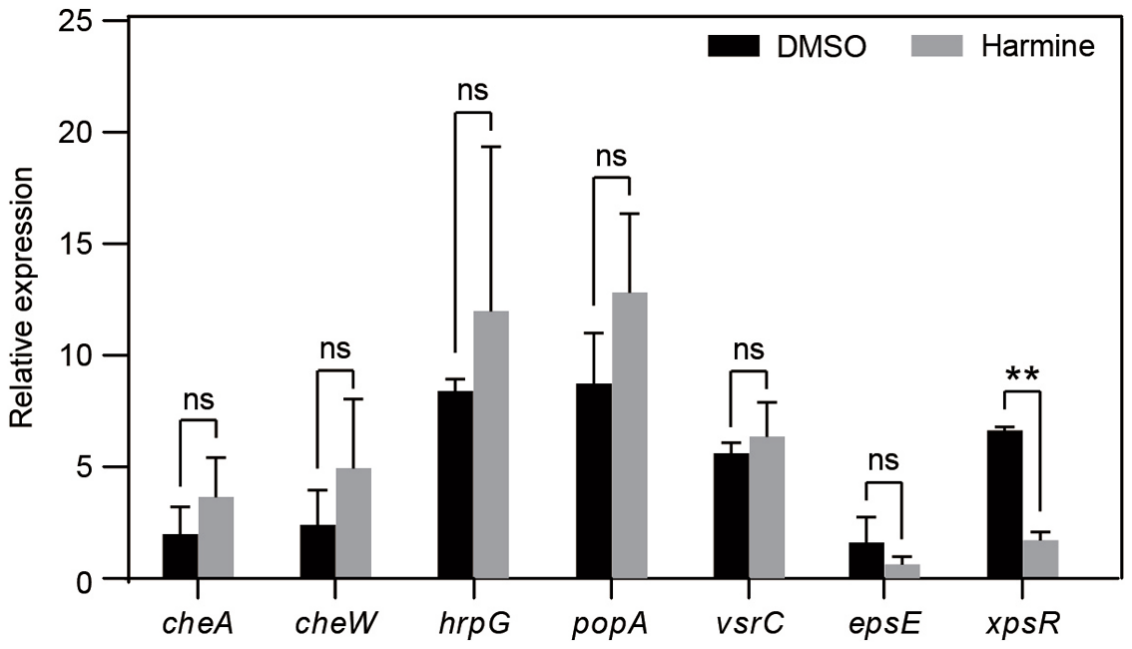
Harmine inhibits the expression of the virulence gene *xpsR*. *R. solanacearum* grown in liquid medium was treated with 80 mg/L harmine for 6 h. Total RNA was extracted and used for qPCR analysis. The expression of candidate genes was normalized to the reference gene *serC*. Data are presented as the mean ± SD (*n* = 3). ns, not significant; **p < 0.05 (Student’s *t*-test).

To gain insights into the preliminary mechanism by which harmine inhibits *R. solanacearum*, we employed qPCR to quantify the expression of several virulence-related genes in the presence and absence of harmine. The analyzed genes included *espE* and *xpsR*, involved in extracellular polysaccharide synthesis, as well as *hrpG* and *popA*, associated with the type III secretion system, and *CheW* and *CheA*, linked to chemotaxis (Han et al., 2021). The results revealed that harmine significantly suppressed the expression of *xpsR*. Furthermore, harmine treatment led to a reduction in *espE* expression, although the difference between DMSO and harmine treatment was not statistically significant. However, the expression of all other tested genes remained unchanged upon harmine treatment (Figure 4). These findings suggest that harmine specifically inhibits genes related to extracellular polysaccharide synthesis.

### Harmine reduces the bacterial wilt disease development

**Figure 5:**
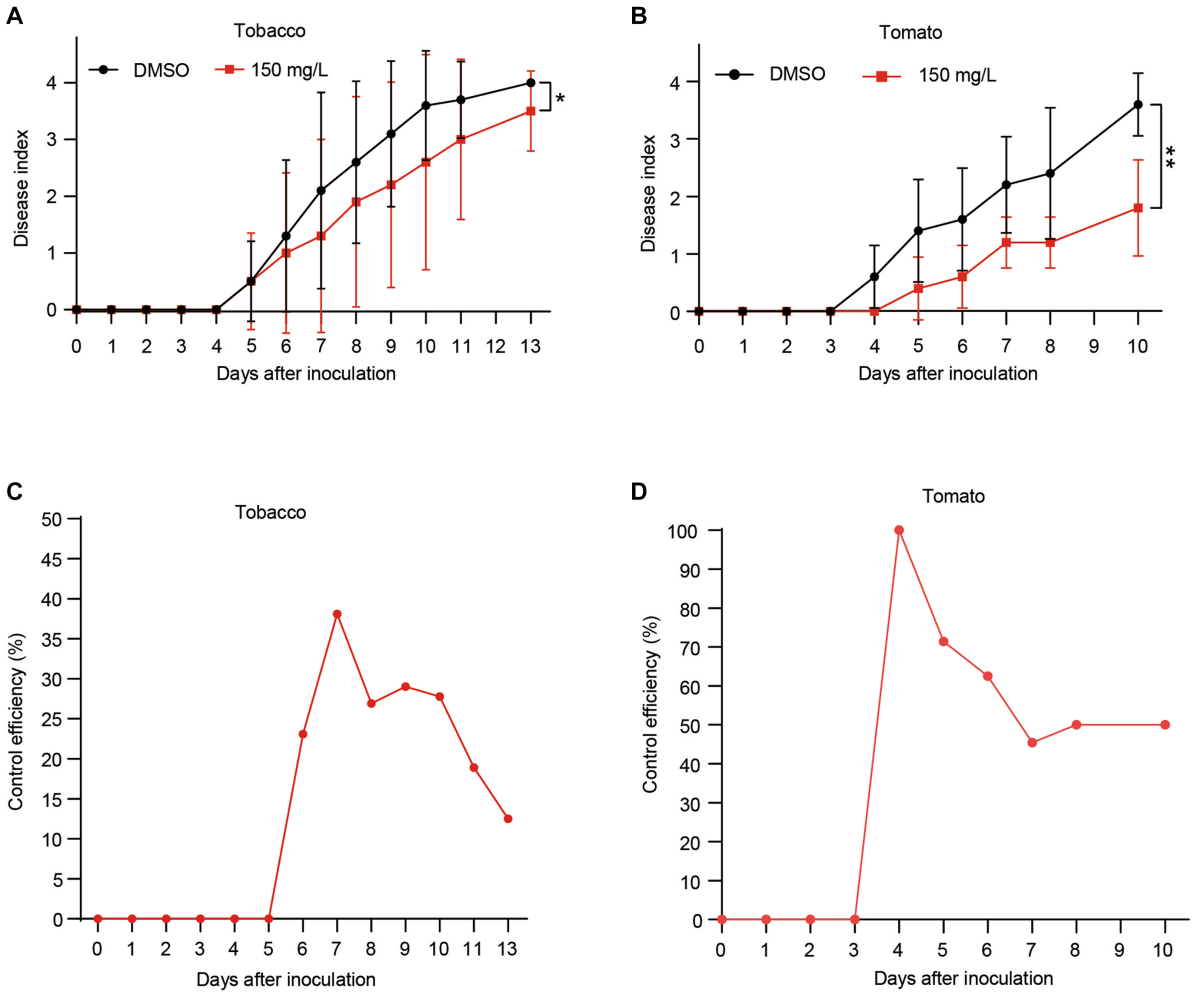
Harmine reduces bacterial wilt disease development in both tobacco and tomato. Four-week-old tobacco **(A)** and tomato **(B)** seedlings were inoculated with 10 mL of bacterial suspension at OD_600_ of 0.1, followed by treating the seedlings with either DMSO or 150 mg/L of harmine. Disease symptoms were recorded daily using a disease index scale ranging from 0 to 4 (0, healthy; 1, 1 to 25% of leaves wilted; 2, 26 to 50% of leaves wilted; 3, 51 to 75% of leaves wilted; 4, 76 to 100% of leaves wilted). Each point represents the mean disease index of 12 plants. *p < 0.05, **p < 0.01 (Student’s *t*-test). **(C,D)** The control efficiency of harmine on tobacco bacterial wilt **(C)** and tomato bacterial wilt **(D)**. The control efficiency was calculated with the following formula: Control efficiency % = (Disease index of DMSO treated seedlings – Disease index of harmine treated seedlings) / Disease index of DMSO treated seedlings × 100.

Having established that harmine exhibits strong antibacterial activity against *R. solanacearum*, reduces its biofilm formation and suppresses *xpsR* expression, we aimed to investigate whether harmine could delay or reduce the development of bacterial wilt disease. Given that *R. solanacearum* infects a wide range of host plants, including Solanaceae crops, we initially examined its effect on tobacco disease development. We first inoculated 4-week-old tobacco seedlings with *R. solanacearum*, followed by treating the seedlings with either DMSO or 150 mg/L. Compared to the DMSO treatment, the 150 mg/L harmine treatment significantly reduced the bacterial wilt disease development in tobacco seedlings (Figure 5A). Next, we assessed the effect of harmine on tomato bacterial wilt development. Similar to tobacco, treatment with 150 mg/L of harmine also resulted in a significant reduction in disease progression in tomato plants (Figure 5B). We further evaluated the control efficiency of harmine against bacterial wilt in both tobacco and tomato. Harmine treatment led to 25 and 50% control efficiency in tobacco and tomato, respectively, at 8 days post-inoculation (Figures 5C,D). These results demonstrate that harmine has the potential to be an effective antibacterial agent for the control of bacterial wilt caused by *R. solanacearum*.

## Discussion

As a soil-borne plant disease, the control of bacterial wilt is extremely challenging, despite that some alternative strategies have been used to control this disease (Jiang et al., 2017). The use of chemical pesticides is the major way for the control of bacterial wilt. Chemical pesticides, while effective in controlling plant diseases in agriculture, can have significant side effects on the environment, human health, and non-target organisms (Tudi et al., 2021; Mansoor and Shad, 2022). Furthermore, pesticide resistance can develop in target pests over time, rendering the chemicals less effective and leading to a need for stronger and more toxic formulations (Ishii, 2006; Shao et al., 2021; Mansoor and Shad, 2022). As a result, there is a growing need to adopt more sustainable and environmentally friendly alternatives to minimize the side effects associated with chemical pesticides. Plant-derived compounds offer several advantages, such as environmentally friendly and lower toxicity to humans, over chemical pesticides, making them an attractive alternative for plant disease control (Sparks and Bryant, 2022). Plant-derived compounds have the potential to revolutionize disease management, providing a greener and more sustainable approach to protect crops while minimizing the negative impacts associated with chemical pesticides. Previous studies have identified several plant-derived compounds, such as alkyl gallates, Lansiumamide B, 7-methoxycoumarin, hydroxycoumarins, coumarins, that show strong inhibitory effect on the growth of *R. solanacearum* (Ooshiro et al., 2011; Li et al., 2014; Yang et al., 2016, 2021a; Han et al., 2021). Some plant-derived compounds are able to suppress the virulence factors (e.g., type III secretion system and biofilm formation) of *R. solanacearum* and thus reduce its pathogenicity on host plants (Yang et al., 2017; Han et al., 2021). However, these studies report the inhibitory effect of single compound on the growth of *R. solanacearum*. Large-scale screening of antibacterial activity of plant-derived compounds against *R. solanacearum* has rarely been reported.

In this study, we conducted a screening of 100 plant-derived compounds to assess their antibacterial activity against *R. solanacearum*. Remarkably, 12 compounds demonstrated strong inhibitory effects on the growth of *R. solanacearum*, with an inhibitory rate exceeding 50% (Table 1). This indicates that a considerable proportion of plant-derived compounds possess potent antibacterial activity against this bacterium. Among the tested compounds, harmine exhibited the most remarkable antibacterial activity compared to the other 11 compounds, with a MIC of 120 mg/L. It is worth noting that this MIC value is slightly higher than the one observed for daphnetin (Yang et al., 2016). Their study assessed the inhibitory effect of daphnetin against *R. solanacearum* using the solid dilution method for 12 h. In contrast, we evaluated the MIC of harmine at 24 h post-treatment. This disparity in testing duration may account for the relatively higher MIC observed for harmine in our study.

Biofilm formation plays a crucial role in bacterial virulence and infection (O’Loughlin et al., 2013). In *R. solanacearum*, biofilms facilitate its ability to infect plants and enhance its colonization in plant roots and stems (Yao and Allen, 2007). Thus, the normal formation of biofilms is essential for the successful infection and proliferation of *R. solanacearum*. Many natural products derived from plants have shown potential in inhibiting the pathogenicity of *R. solanacearum* by restricting biofilm formation (Wu et al., 2015; Yang et al., 2017; Han et al., 2021). This study found that harmine can effectively inhibit the formation of biofilms by *R. solanacearum*. In previous research, the minimum concentration required to inhibit biofilm formation generally correlated with the MIC of the compound. However, in our study, we observed that the lowest concentration of harmine needed to inhibit biofilm formation was significantly lower than the MIC, indicating that low concentrations of harmine are still able to suppress biofilm formation by *R. solanacearum*.

The accumulation of extracellular polysaccharides is a key trigger for wilting symptom development in plants infected with *R. solanacearum* (Saile et al., 1997). This accumulation facilitates the rapid wilting of infected plants (Genin and Denny, 2012; Lowe-Power et al., 2018). Studies have shown that ginger extract can inhibit the growth of *R. solanacearum* and significantly reduce the production of extracellular polysaccharides by the bacterium (Zhang et al., 2022). After treating *R. solanacearum* with harmine *in vitro*, we observed a significant decrease in the expression of the key transcription factor *xpsR*, which regulates the biosynthesis of extracellular polysaccharides (Huang et al., 1995). Previous research involving the plant growth regulator silicon demonstrated its ability to inhibit the expression of the *xpsR* gene and the secretion of extracellular polysaccharides in *R. solanacearum*, thereby reducing its pathogenicity (Wang et al., 2022). This suggests that harmine reduces the pathogenicity of *R. solanacearum* by inhibiting the expression of the *xpsR* gene. Notably, the expression of *epsE*, a downstream gene involved in extracellular polysaccharide biosynthesis, did not show a significant suppression by harmine, although a decrease in expression was observed. This could be attributed to the fact that *xpsR* regulates the expression of multiple genes, and its inhibitory effect on a single gene may be attenuated.

Our study found that harmine did not inhibit the expression of several other virulence-related genes tested, such as those associated with the type III secretion system or chemotaxis. We speculate that this discrepancy may be due to the nutrient-rich medium used in our study, as *R. solanacearum* may only express type III secretion system associated genes in nutrient-poor minimal medium (Genin et al., 1992; Zhang et al., 2011). Interestingly, harmine was recently shown to inhibit the type III secretion system of *Salmonella enterica* serovar Typhimurium (Shi et al., 2022). *S. enterica* serovar Typhimurium is an animal pathogenic bacterium. Given the differences of the type III secretion system proteins between animal and plant pathogenic pathogens, it is conceivable that harmine targets the type III secretion system of specific pathogens instead of a broad range of pathogens. Therefore, harmine may specifically target certain pathways, such as *xpsR*, rather than affecting multiple aspects of *R. solanacearum*’s virulence.

In summary, this study identified several plant-derived compounds as potent antibacterial agents against *R. solanacearum*, with harmine exhibiting the strongest activity, with a MIC of 120 mg/L. Harmine significantly inhibited *R. solanacearum* biofilm formation and downregulated the expression of certain virulence-related genes. Moreover, harmine effectively suppressed the development of tobacco and tomato bacterial wilt disease. These findings suggest that plant-derived compounds, like harmine, hold tremendous potential for controlling the devastating bacterial wilt disease caused by *R. solanacearum*.

## Data availability statement

The raw data supporting the conclusions of this article will be made available by the authors, without undue reservation.

## Author contributions

HX: Writing – original draft, Investigation. YH: Writing – original draft, Investigation. RW: Investigation, Writing – review & editing. XT: Investigation, Writing – review & editing. JC: Investigation, Writing – review & editing. S-xL: Conceptualization, Writing – review & editing. LJ: Conceptualization, Funding acquisition, Writing – original draft. DW: Conceptualization, Funding acquisition, Writing – original draft.

## Funding

The author(s) declare financial support was received for the research, authorship, and/or publication of this article. This work was supported by the Natural Science Foundation of Chongqing (CSTB2022NSCQ-MSX0813), the Natural Science Foundation of Hunan (2021JJ40058 and 2023JJ60473), the Young Elite Scientists Sponsorship Program by CAST (grant no. 2021QNRC001), the National Natural Science Foundation of China (82104055), the China Postdoctoral Science Foundation (2020 M671163), and the Hunan Provincial Department of Education Science Research Project (22B0396).

## Conflict of interest

The authors declare that the research was conducted in the absence of any commercial or financial relationships that could be construed as a potential conflict of interest.

## Publisher’s note

All claims expressed in this article are solely those of the authors and do not necessarily represent those of their affiliated organizations, or those of the publisher, the editors and the reviewers. Any product that may be evaluated in this article, or claim that may be made by its manufacturer, is not guaranteed or endorsed by the publisher.
